# Transfer learning with chest X-rays for ER patient classification

**DOI:** 10.1038/s41598-020-78060-4

**Published:** 2020-12-01

**Authors:** Jonathan Stubblefield, Mitchell Hervert, Jason L. Causey, Jake A. Qualls, Wei Dong, Lingrui Cai, Jennifer Fowler, Emily Bellis, Karl Walker, Jason H. Moore, Sara Nehring, Xiuzhen Huang

**Affiliations:** 1 Center for No-Boundary Thinking (CNBT) at Arkansas State University, The Joint Translational Research Lab of Arkansas State University, St. Bernards Medical Center, Jonesboro, AR 72467 USA; 2 The Internal Medicine Residency Program, St. Bernards Medical Center, 225 E Jackson Ave, Jonesboro, AR 72401 USA; 3 Department of Computer Science and Molecular Biosciences Program, Arkansas State University, Jonesboro, AR 72467 USA; 4 Ann Arbor Algorithms, Ann Arbor, MI 48103 USA; 5 Institute for Biomedical Informatics, University of Pennsylvania, Philadelphia, PA 19104 USA; 6 Department of Mathematics and Computer Science, University of Arkansas At Pine Bluff, Pine Bluff, AR 55455 USA; 7 Arkansas Biosciences Institute, Arkansas State University, Jonesboro, AR 72467 USA

**Keywords:** Computational biology and bioinformatics, Diseases, Medical research, Mathematics and computing

## Abstract

One of the challenges with urgent evaluation of patients with acute respiratory distress syndrome (ARDS) in the emergency room (ER) is distinguishing between cardiac vs infectious etiologies for their pulmonary findings. We conducted a retrospective study with the collected data of 171 ER patients. ER patient classification for cardiac and infection causes was evaluated with clinical data and chest X-ray image data. We show that a deep-learning model trained with an external image data set can be used to extract image features and improve the classification accuracy of a data set that does not contain enough image data to train a deep-learning model. An analysis of clinical feature importance was performed to identify the most important clinical features for ER patient classification. The current model is publicly available with an interface at the web link: http://nbttranslationalresearch.org/.

## Introduction

In this study, we focused on acute respiratory distress syndrome (ARDS) in an emergency room (ER) setting. There are many etiologies of acute dyspnea (or shortness of breath), but our model focuses on identifying two major categories: cardiac and infectious. Upon admission to a hospital emergency department, attending physicians must quickly determine which category the patient falls into. Typically, these patients receive a suite of common clinical panels as well as a chest X-ray image early in the diagnostic process. We have developed a machine learning model capable of assisting ER physicians with categorizing the acute dyspnea given clinical values alone, or in conjunction with an X-ray image if one is available.

**Figure 1:**
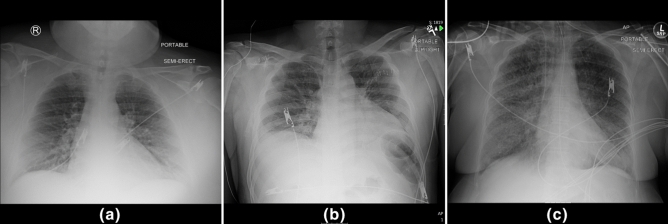
Examples of chest X-ray images where the ground truth label is (**a**) neither infection nor cardiac label, (**b**) infection label, (**c**) cardiac label.

Cardiac causes include etiologies of dyspnea secondary to a misfunction in the heart, including acute coronary syndrome, acute heart failure, arrythmias, and valvular disease^1^. These diseases do not benefit from antibiotic therapy. Infectious causes of acute dyspnea include both pneumonia, an infectious process primary to the lungs, and sepsis, a systemic response to an infection anywhere in the body that can impair function in a variety of organs^1^. When severity is life-threatening, suspicion of these disease processes require empiric antibiotics. Antibiotics have adverse effects in 1 out 5 patients^2^. Though these adverse effects often include direct side-effects from the drugs themselves, antibiotics can also produce adverse effects through their interactions with microorganisms^2^. Our model represents a step toward more rapidly confirming or excluding infectious etiology, and thus reducing the unnecessary empiric prescription of antibiotics. Other causes of acute dyspnea may fall into neither of these categories^1^, or a patient may have acute dyspnea with both cardiac and infectious contributions. See Fig. 1 for examples of X-ray images for patients whose correct labeling should be (a), “infection” label (b), and “cardiac” label (c).

Recent advances in machine learning have shown that it can be a valuable tool for aiding in medical planning. The CheXNet^3^ model was able to accurately identify 14 categories of abnormalities in chest X-ray images. Deep learning techniques have shown promise for automated detection and diagnosis of lung cancer^4–7^, breast cancer^8,9^, skin cancer^10–12^, and other diseases. Most of these approaches use deep neural networks^13^ especially convolutional neural networks^14,15^. In data domains outside imaging, gradient boosted trees and the XGBoost^16^ model in particular, have been used to solve a wide variety of problems where the inputs may include variables of differing types^17–20^.

Current models for evaluating ARDS are typically limited to scoring tools for use by physicians, machine learning tools for predicting the incidence of ARDS, machine learning tools for predicting the severity or ARDS, and machine learning tools that discover distinct phenotypes in ARDS. Multiple scores for use in clinical medicine have been produced, such as the modified ARDS prediction score (MAPS)^21^. Other scores exist, but with the Berlin definition of ARDS, some researchers question the continued need for these clinical scoring systems^22^. Many of the machine learning models relating to ARDS are focused on predicting the incidence of ARDS^23^ or the severity of ARDS^24^. These models accomplish a different task than our model, which focuses on the cause of ARDS, not on identifying its presence or severity. The most similar models in scope to ours are the models that focus on discovering distinct phenotypes of ARDS from clinical data^25,26^. They are similar in that they distinguish between multiple sub-types of ARDS. However, the sub-types distinguished in these models are learned rather than pre-defined. Our model distinguishes between two pre-defined subclasses of ARDS with special clinical significance: Those with an infectious etiology and those with a cardiac etiology.

Our model makes use of both deep neural networks and XGBoost for examining images and clinical data, respectively, and the combination of the two is handled by extracting image features via a deep neural network and performing classification using XGBoost. Specifically, our model performs independent binary classification against two categorical labels (infection, cardiac), giving four possible labelings: neither label applies, one of the labels applies, or both labels apply. The feature extraction is performed by a deep convolutional neural network (CNN) model named CheXNet^3^, which was originally designed to predict 14 categories of abnormalities in chest X-ray images, but did not focus specifically on ARDS. By utilizing an output vector from CheXNet as input for our additional classifier, we are able to transfer the high-level latent representation of the X-ray’s image features and specialize the final classifier using a limited amount of training utilizing a general form of transfer learning^27^.

## Results

### Performance in infection labeling task

**Figure 2:**
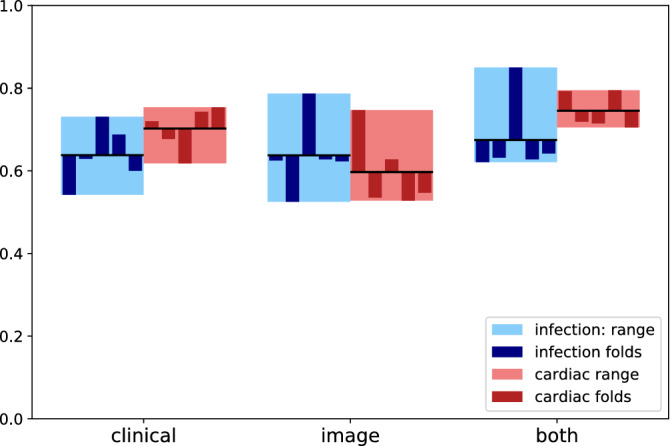
Summary of fivefold cross validation results. Infection range (light)/fold (dark) performance is shown in blue. Cardiac range (light)/fold (dark) performance is shown in red. Black horizontal bars denote the mean across all folds.

On the “infection” labeling task, our model achieved an average accuracy of 63.8% (SD = 7.4%) using the clinical features alone. Using image (CheXNet) features only, average accuracy was 63.8% (SD = 9.4%). When both types of features were combined, the average performance was 67.5% (SD = 9.8%), which was a modest (3.7%) improvement over either of the single-modality models alone. Figure 2 shows a plot representing the accuracy values over each of the five folds, along with their range and mean (as well as the same information for the “cardiac” labels, discussed in the next section). For comparison, the same cross validation was performed using a logistic regression model and a k-Nearest Neighbors model (k = 5). Table 1 summarizes the results for all five folds of the primary model, as well as the average performance for all three models on the “infection” task. It can be seen that although the k-NN model performs quite well when only clinical features are considered, the primary (XGBoost) model is best able to aggregate features from both modalities.

**Table 1:** Five-fold cross validation results for “infection” label, showing clinical-only, image-only, and combined performance.

Fold	Clinical	Image	Both
1	0.542	*0.625*	0.621
2	0.629	0.525	*0.632*
3	0.731	0.787	*0.85*
4	*0.688*	0.628	0.628
5	0.6	0.623	*0.642*
Avg (SD)	**0.638 (0.074)**	0.638 (0.094)	***0.675 (0.098)***
Logistic reg	0.535 (0.105)	***0.678 (0.126)***	0.570 (0.116)
k-NN (k = 5)	*0.637 (0.030)*	0.528 (0.128)	0.637 (0.030)

Each fold (1–5) is shown, followed by average performance. Top average performance for each feature type is shown in bold, best result for each row in italics. Average performance for a logistic regression model and k-NN model (k = 5) shown in last two rows for comparison.

### Performance in cardiac labeling task

On the “cardiac” labeling task, our model achieved an average accuracy of 70.2% (SD = 5.6%) using the clinical features alone. An accuracy of 59.5% (SD = 9.3%) was achieved using the image (CheXNet) features alone. The combined clinical and image features improved the accuracy 4.3% to 74.5% (SD = 4.5%). Figure 2 shows a plot summarizing our model’s accuracy in each of the five folds, along with their range and mean for both the “infection” and “cardiac” tasks. For comparison, the same cross validation was performed using a logistic regression model and a k-Nearest Neighbors model (k = 5). Table 2 summarizes the results for all five folds of the primary model, as well as the average performance for all three models on the “cardiac” task. As with the “infection” task, the primary (XGBoost) model was best able to aggregate features from both the clinical and image modalities.

**Table 2:** Five-fold cross validation results for “cardiac” label, showing clinical-only, image-only, and combined performance.

Fold	Clinical	Image	Both
1	0.72	0.747	*0.793*
2	0.677	0.535	*0.719*
3	0.618	0.628	*0.715*
4	0.743	0.528	*0.795*
5	*0.754*	0.547	0.705
Avg (SD)	0.702 (0.056)	0.597 (0.093)	**0.745 (0.045)**
Logistic reg	0.604 (0.059)	***0.656 (0.122)***	0.637 (0.048)
k-NN (k = 5)	*0.677 (0.077)*	0.577 (0.145)	0.677 (0.077)

Each fold (1–5) is shown, followed by average performance. Top average performance for each feature type is shown in bold, best result for each row in italics. Average performance for a logistic regression model and k-NN model (k = 5) shown in last two rows for comparison.

### SHAP feature importance analysis

**Figure 3:**
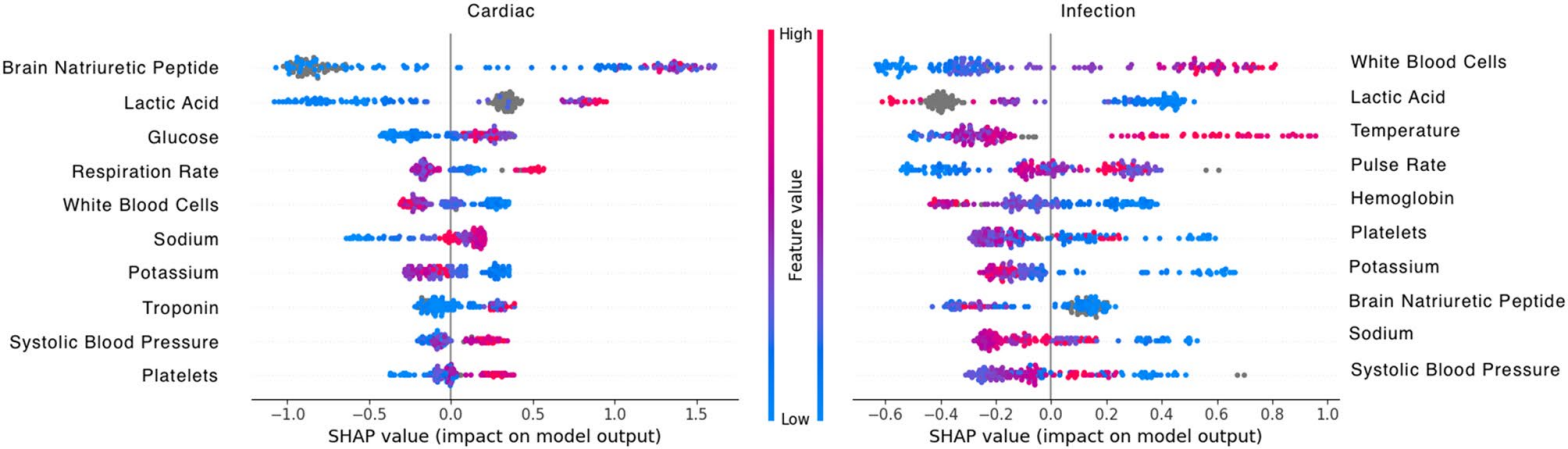
SHAP TreeExplainer Feature importance plot for the top ten clinical features for the “cardiac” task (left) and the “infection” task (right). Color denotes feature magnitude, X-axis shows SHAP value, feature names are shown to the side of each row.

**Figure 4:**
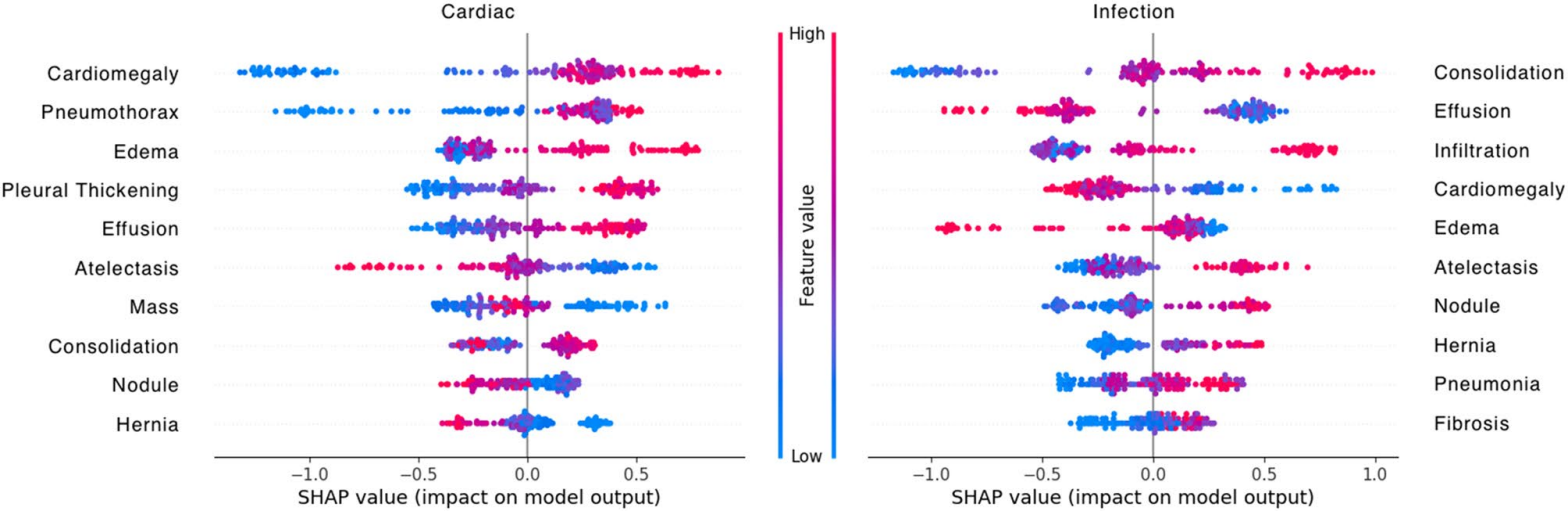
SHAP Feature importance plot for the top ten image features for the “cardiac” task (left) and the “infection” task (right). Color denotes feature magnitude, X-axis shows SHAP value, feature names are shown to the side of each row.

The SHAP “TreeExplainer” algorithm was used to determine the most important features in both the clinical and imaging modalities. All 171 examples were used for the SHAP analysis. Figure 3 shows the SHAP feature importance analysis for clinical features on both the “cardiac” and “infection” labeling task. Figure 4 shows the SHAP feature importance analysis for image features on both the “cardiac” and “infection” labeling task. The image feature names correspond to the categorical label as defined by CheXNet^3^. Each point in the figure is a feature value of a particular training example. The color of the point represent the feature value and the X-axis position of the point is its SHAP value. The features are ranked by the sum of SHAP value magnitudes over all samples. The top ten ranked features are shown (in ascending order), with feature names shown to the side of each plot row.

#### SHAP analysis, infection task

SHAP analysis of feature importance for prediction of infection was consistent with current medical knowledge. White blood cells count is expected to rise in response to infection^28^ and was found to be the most important feature for determining the presence of infection. Similarly, fever (elevation of temperature) was found to be predictive of infection.

SHAP analysis of imaging features for prediction of infection is also consistent with clinical knowledge. Consolidation and infiltration can both be radiographic features of a pneumonia^29^.

#### SHAP analysis, cardiac task

SHAP analysis of feature importance for prediction of a cardiac cause of respiratory distress also followed a reasonable pattern (see Figs. 3, 4). Brain natriuretic peptide (B-NP) was rated by the model as its most important feature for predicting a cardiac cause of infection. Normal values for B-NP have been shown to have a high negative-predictive value for heart failure^30^ and are used to diagnose exacerbation of existing heart failure^31^. Blood glucose levels are not directly associated with heart failure, but the model may be looking for associated diabetes mellitus. This common disease is an important risk factor for heart disease^32^. Increases in respiratory rate can be caused by heart failure exacerbations^33^, so it makes sense that this would be an important predictive feature.

The model’s evaluation of imaging features for cardiac causes are less intuitive. The model highly values cardiomegaly, effusion, and edema as predictive of a cardiac cause. All these radiographic findings can be present in heart failure^30,33^. However, the model’s use of the pneumothorax and pleural thickening features as predictive of heart failure, do not make clinical sense. The model may be using these features to evaluate for the presence of Kerley B lines. These lines are commonly associated with heart failure and are adjacent to the pleura^34^.

### Feature comparison

We have conducted feature comparison analysis. Table 3 shows the top five clinical features for each labeling task. The top features were determined by summarizing all absolute values of SHAP values by features and then ranking features based on the sum. Table 4 shows the top five clinical panels for each labeling task. The top panels were determined by further summarizing and ranking the per-feature SHAP value magnitudes. A panel with more components is potentially favored in ranking as more values are added together. The single-component feature B-NP ranks 1st in the Cardiac experiment suggesting that it is a very strong indicator. Table 5 shows the top five imaging features, labeled by their corresponding CheXNet label^3^ for each labeling task. The top features were determined by SHAP analysis as previously described.

**Table 3:** Top five clinical features by labeling task.

Rank	Infection	Cardiac
1	White_Blood_Cells	Brain_Natriuretic_Peptide
2	Lactic_Acid	Lactic_Acid
3	Temperature	Glucose
4	Pulse_Rate	Respiration_Rate
5	Hemoglobin	White_Blood_Cells

**Table 4:** Top five clinical panels comparison.

Rank	Infection	Cardiac
1	Vitals (6)	B-NP (1)
2	CBC (3)	BMP (7)
3	BMP (7)	Vitals (6)
4	ABG (4)	Lactic Acid (1)
5	Lactic Acid (1)	CBC (3)

The number of features in each panel is shown in parentheses.

**Table 5:** Top five image features comparison.

Rank	Infection	Cardiac
1	Consolidation	Cardiomegaly
2	Effusion	Pneumothorax
3	Infiltration	Edema
4	Cardiomegaly	Pleural_Thickening
5	Edema	Effusion

#### Feature importance

As seen in Table 3, the model ranks lactic acid measurements as its second most important feature for both infectious and cardiac causes of respiratory distress. Lactic acidosis is usually caused by global hypoperfusion which could be secondary to cardiac (cardiogenic shock) or infectious (sepsis) causes^35^. This suggests that, in our dataset, patients with cardiac causes of acute respiratory distress are more likely to also present with lactic acidosis than those with infectious causes, or that they are likely to develop lactic acidosis sooner. This makes clinical sense as an infection in the lungs need not have systemic effects to cause respiratory distress whereas heart failure is expected to have systemic effects.

The model seems to view the classifications of infection and cardiac causes of acute respiratory distress as somewhat dichotomous. For instance, high values of lactic acid are associated with cardiac causes and low values are associated with infectious causes, and this value is ranked as the second most important for both classifications. Similarly, white blood cell count is the most important laboratory value for infection and the fifth most important for cardiac, with high values associated with infection and low values associated with cardiac.

As seen in Table 5, we observe imaging features shared between the infection and cardiac classifications, with edema, cardiomegaly, and effusion in the top 5 features for both categories. Even though effusion can sometimes be associated with complicated pneumonia^33^, the model treats all of these features as favoring cardiac causes while disfavoring infectious causes, reinforcing the model’s overall dichotomous view of these disease processes.

## Summary and discussion

### Current performance of the model

We have shown that a combination of imaging and clinical features improved overall performance of XGBoost on predicting both infectious and cardiac causes of acute respiratory distress. For the infection labeling task, the combined model performed best in 3 of the 5 cross validation folds and performed slightly better on average. In this task, the performance was only marginally better than clinical features alone, perhaps due to the higher variance in visual presentation of infectious conditions. In the cardiac labeling task, the combined model performed best in 4 out of 5 folds. Interestingly, in this task the imaging model alone significantly underperformed the clinical model, but the image features provided a larger overall improvement when added to the clinical features than we saw on the infection labeling task. The combination seems to improve the consistency of XGBoost on prediction of cardiac causes.

For comparison, we presented results obtained from a logistic regression model and a k-Nearest Neighbors model (k = 5). Both baseline models were from the Python SciKit-Learn library. These results can be seen in the last two rows of Tables 1 and 2. It must me mentioned that the baseline models were only be able to make predictions on the (preprocessed) “clinical” feature set directly. We included the “image” and “both” feature combinations for completeness, but to do so, the image features required the same deep learning feature extraction stage (CheXNet) used with our primary model. As such these two applications represent alternate configurations of our primary approach. These simpler models each performed reasonably well on one of the feature types: k-NN tended to work reasonably well on clinical features, while the linear logistic regression tended to work well on the image-only feature set. This could be due to the relatively small dimensionality of the image feature vector. Neither of the alternative models showed much improvement when combining features from the two modalities, suggesting that the XGBoost model is more robust when multi-modal features are present.

### Possible improvements and future research

The main limitation on this model’s current performance was the relatively small number of example cases. The dataset of 171 patients was far below an ideal number for training. However, we are continuing to expand our dataset. As our dataset grows, we expect significant performance improvements. We are also exploring new image model formulations that make use of “localization” annotations we were able to collect on our dataset. These annotations should allow us to provide addition feedback to the image model to serve as a forcing function for an attention mechanism. With an updated model and by expanding our dataset to hundreds of cases, we expect accuracy to make significant improvements in performance.

In our next phase of research, we will allow our collaborating resident physicians to apply the model to new patients and help guide decision making. This will allow us to evaluate the model’s efficacy in improving patient outcomes and reducing antibiotic use. Additionally, this project began before the recent SARS-CoV-2 pandemic. As we move forward with development, we will explore upgrading the model to include a SARS-CoV-2 specific classification with COVID-19 patients’ data. This would allow physicians to use the same software to diagnose cases of SARS-CoV-2 pneumonia. We expect our model to be able to perform this task with a high accuracy as other research teams have had success with this problem^36^. This would also support our goal of improving antibiotic stewardship among physicians as SARS-CoV-2 pneumonia does not benefit from antibiotic therapy^37^.

## Methods

### Statement

Statement regarding informed consent. Informed consent regarding this research was waived by the St. Bernards Medical Center’s Institutional Review Board (IRB). This study was approved by the St. Bernards Medical Center’s Institutional Review Board (IRB). The research results of this paper are related to Part One of the study of the Translational Research Lab. For Part One of the study, we are completely de-identifying the patient, there is no risk to the patient, and it would be impractical to obtain consent, given the number of charts to be reviewed for data collection. Confidentiality breach is the only risk to the patient, and we would be increasing that risk by obtaining signed informed consent. No interventions were undertaken during this portion. For our future work on Part Two of this study, the patients selected will be patients that are seen in conjunction with the Internal Medicine Residency Program (IMRP) resident assigned to the ER for that month, who will be responsible for obtaining consent, and are subsequently admitted to SBMC for further care as an inpatient.

All methods were carried out in accordance with relevant guidelines and regulations.

### Clinical data preprocessing

The dataset contains clinical data of 188 patients and chest X-ray images of 171 patients. Each patient has two Boolean classification labels: cardiac and infection, of which both can be true.

We used the clinical and image data of the 171 patients who had both for evaluation. The clinical data were hand-entered by a group of residents on rotation and contained some data entry errors that required careful cleaning before it could be used. In total, we were able to utilize 23 features from the clinical panels as described below.

The complete blood count (CBC) with differential column always contained 3 or 4 values. Based on what CBC with diff reports, conventional notation, and their ranges, these values were white blood cell count, hemoglobin, hematocrit, and platelets, respectfully. When three values were present, hematocrit was always assumed to be missing based again on ranges and conventions. Of note, hematocrit should be able to be calculated from hemoglobin and is somewhat of a “redundant” value. After cleaning, hematocrit was excluded from the final analysis due to a preponderance of missing values.

The basic metabolic profile (BMP) test reports sodium, potassium, chloride, bicarbonate, blood urea nitrogen, creatinine, and glucose. By convention, they are reported in this order. The original dataset included some missing values in the BMP report. We identified which values were missing based on the positions and ranges of the values present compared to typical ranges for corresponding components of the BMP.

The column for brain natriuretic peptide (B-NP) always contained a single value. Where a real number was present, the value was kept as is. Otherwise, it was given an appropriate sentinel value to represent “missing”.

The first troponin measurement was represented as a continuous (real number) value, but sometimes contained values that could be directly interpreted as a real number. Values such as “ < 0.012” were given the sentinel value “0” for “undetectable.” Multiple values were sometimes given, documenting the trend of multiple troponin measurements. In these cases, only the first measurement was kept.

The procalcitonin measurements contained too many missing values to be used in the final analysis, so it was excluded.

The lactic acid value was measured as a continuous (real number) value. All instances containing a value that could be directly interpreted as a real number were kept. All other values were marked as “missing”.

The vital signs column usually contained 6 values in the following format: Temperature; Pulse Rate; Systolic Blood Pressure/Diastolic Blood Pressure; Respiration Rate; Pulse Oximetry. Real number values were recorded without lettering or comments. Though pulse oximetry is typically recorded as a percentage, we converted it to a real number in the range [0,1]. The residents recording these measurements were not consistent with the ordering of these values. The overwhelming common alternative format transposed the blood pressure and pulse rate values. Missing values were identified using typical ranges of these values, the order of the values, and the fact that blood pressure values are always expressed as x/y with x > y. Information about the patient’s use of supplemental oxygen was not kept.

The arterial blood gas column was the most problematic. There were usually 4 or 5 values: Arterial pH, arterial pressure of CO2 (PCO2), arterial bicarbonate (bicarb), arterial pressure of O2 (PO2), and pulse oxygenation (SpO2) at time of blood draw. The residents recording these measurements were least consistent following the conventional order for this column. The conventional order of pH PCO2, bicarb, PO2, SpO2 was assumed unless the values were outside the typical range. However, interpretation was limited as the typical and possible ranges of PCO2 and PO2 overlap significantly. Though recommended for proper interpretation, information on SpO2 and patient supplemental oxygen utilization were not included.

The logistic regression and k-NN models required that all missing values (represented by NaN, or “not a number” values initially) were replaced with a numeric sentinel value. This additional step was not necessary for the XGBoost primary model.

### CheXNet-based image features

Training a deep convolutional neural network model typically requires a large number of images (200—1000 images per class)^38,39^. In this dataset we had 171 images, which is too few to attempt training from scratch. Instead, we opted to use a pre-trained neural network model from a similar application area as a feature extractor. CheXNet^3^ is a 121-layer convolutional neural network trained on the NIH ChestX-ray14^40^ dataset, consisting of 100,000 frontal X-ray images with 14 disease labels. We used the open source PyTorch implementation of CheXNet^3^ available at (https://github.com/arnoweng/CheXNet) with the pre-trained weights provided.

We utilized the 14 output class scores produced by the output stage of the pre-trained CheXNet model as 14 image features, and performed testing to determine whether adding these image features to the clinical features could improve classification accuracy. The CheXNet output scores are real number values in the range [0,1] and were originally interpreted as the probability that the input chest X-ray image should be labeled with the corresponding medical condition. We re-interpreted the values as a 14-dimensional feature vector which was concatenated to our preprocessed clinical features. The rationale was that this feature vector contains a high-level encoding of the medically relevant abnormalities observed in the X-ray image.

### Model training and evaluation

We used XGBoost^16^, an open-source implementation of gradient boosted decision trees. The model was trained and evaluated on the dataset using fivefold cross-validation. We used the following parameter settings: ‘n_estimators’ = 1000, ‘learning_rate’ = 0.01, ‘max_depth’ = 2, ‘subsample’ = 0.50, ‘colsample_bytree’ = 0.60, ‘objective’:‘binary:logistic’. For comparison, we also evaluated a logistic regression model and a k-Nearest Neighbors model where $k$ was set to 5 (the default for the SciKit-Learn implementation we used). The same fivefold cross validation splits were utilized across all three model types.

Experiments utilizing only the “clinical” feature set were conducted by providing the preprocessed clinical features as the input of the classification model. The logistic and k-NN model had missing (“NaN”) values replaced with a valid numeric sentinel value; the XGBoost model did not. Experiments utilizing only the “image” feature set were presented with the 14-dimensional feature vector from the CheXNet output stage as their input. Experiments utilizing “both” feature sets were presented with the preprocessed clinical features concatenated with the 14-dimensional feature vector from CheXNet. Missing clinical values were replaced as described above for the logistic regression and k-NN models.

### SHAP analysis of feature importance

SHAP (SHapley Additive exPlanations) is a game theoretic approach to machine learning model explanation^41^. Lundberg et al. examined several contemporary algorithms for determine feature importance and showed that they belonged to the same class of measures, then unified them into the SHAP framework^42^. The SHAP analysis computes the Shapley value to each individual feature of a training sample. The Shapley value, which is a concept from game theory, represents a feature’s responsibility for a change in the model’s output. The features themselves are viewed as cooperating participants in a game with the goal of solving the machine learning problem. The Shapley value represents the degree to which the individual feature influences the coalition. The sum of the magnitudes of the SHAP values across training examples provides a direct measure of the importance of a feature^42^. We used the Python implementation of SHAP^43^. The “TreeExplainer” algorithm provided by the SHAP library was used for the analyses presented here.

### Ethics

This study was approved by the St. Bernards Medical Center’s Institutional Review Board (IRB).

## Data Availability

The clinical data and chest x-ray image data for this study were collected and prepared by the residents and researchers of the Joint Translational Research Lab of Arkansas State University (A- State) and St. Bernards Medical Center (SBMC) Internal Medicine Residency Program. As data collection is on-going for the project stage-II of clinical testing, raw data is not currently available for data sharing to the public.

## Code availability

We have published the current version of this model on the Internet for evaluation purposes. Physicians will be able to enter the data and receive a prediction through a web interface for research purposes. Eventually, our goal is to aid emergency room clinicians in planning treatment strategies, although clinical evaluation and approval is required before it can be used as a diagnostic and planning tool. It is available at: http://nbttranslationalresearch.org/.
